# A follow-up study of Opisthorchis viverrini infection after the implementation of control program in a rural community, central Thailand

**DOI:** 10.1186/1756-3305-6-188

**Published:** 2013-06-20

**Authors:** Picha Suwannahitatorn, Saranapoom Klomjit, Tawee Naaglor, Paanjit Taamasri, Ram Rangsin, Saovanee Leelayoova, Mathirut Mungthin

**Affiliations:** 1 Department of Parasitology, Phramongkutklao College of Medicine, 315 Ratchawithi Road, Ratchathewi, Bangkok 10400, Thailand; 2 4th year Medical Student, Phramongkutklao College of Medicine, 315 Ratchawithi Road, Ratchathewi, Bangkok 10400, Thailand; 3 Department of Military and Community Medicine, Phramongkutklao College of Medicine, 315 Ratchawithi Road, Ratchathewi, Bangkok 10400, Thailand

**Keywords:** Opisthorchis viverrini, Incidence, Risk factors, Koi pla, Qualitative study, Central Thailand

## Abstract

**Background:**

Opisthorchis viverrini infection is still one of the public health problems in Thailand. Our recent cohort study conducted in a rural community in central Thailand showed that the incidence rate of O. viverrini infection in 2002–2004 was 21.6/100 person-years. Conventional control activities including case diagnosis and treatment, hygienic defecation promotion and health education focusing on avoiding raw fish consumption was implemented. This study aimed to re-assess the status of infection after implementation of intervention programs, using both quantitative and qualitative methods in 2007–2009.

**Methods:**

A prospective cohort study was conducted to evaluate the incidence and risk factors of O. viverrini infection. Stool examination methods including wet preparation, Kato and formalin-ethyl acetate concentration technique were performed for the detection of O. viverrini eggs. A standardized questionnaire was used to assess risk behavior. In addition, qualitative information was collected from both O. viverrini negative and positive villagers using focus group discussions.

**Results:**

The incidence of O. viverrini infection was 21.4/100 person-years. Consumption of chopped raw fish salad, Koi pla and age 60 years and older were independently associated with O. viverrini infection, similar to our previous study. Findings from the qualitative study, indicated that inadequate knowledge, misbeliefs, and social and cultural mores were important factors leading to the maintenance of risk behaviors. Moreover, unhygienic defecation and insufficient diagnosis and treatment were found to facilitate O. viverrini transmission.

**Conclusion:**

Although the conventional control program had been used in the study population, the incidence of O. viverrini infection remained the same. Precise and regular health education and promotion targeting the main risk factor, Koi pla consumption, improving diagnosis and treatment, and promoting hygienic defecation should be used in the prevention and control program.

## Background

Human liver fluke infection caused by Opisthorchis viverrini, is prevalent in Southeast Asia [1-5]. O. viverrini is a pathologically important food-borne trematode which infects the hepato-biliary system [6-8]. Chronic O. viverrini infection is related to cholangiocarcinoma for which the International Agency for Research on Cancer (IARC) has declared O. viverrini to be a carcinogen to humans [6,7].

Global estimation of people infected with O. viverrini is ~9 million, most of which are in Thailand and Lao PDR [3,9,10]. In 2001, The National Health Survey in Thailand showed various distributions of infection [11,12]. In Thailand, prevalence was high in the north (19.3%) and the northeast (15.7%) while the prevalence in central Thailand was much lower at 3.8% [7]. The incidence of cholangiocarcinoma in Khon Kaen, a province in northeastern Thailand, ranged from 93.8 to 317.6 per 100,000 person-years from 1990 to 2001 [7]. The concentrated distribution of O. viverrini in the north and northeast regions corresponds with local natural intermediate hosts and traditional eating habits [6,7]. Although the life cycle of O. viverrini is complex, involving two intermediate hosts, the consumption of uncooked food containing parasites at the infective stage leads to the infection. The first intermediate host is the Bithynia snail and the second intermediate host comprises freshwater fish, i.e. Cyprinoid family [2,13]. By consuming uncooked freshwater fish, people will be infected with its infective stage parasites called metacercariae. In local areas of north and northeast Thailand, preparations of raw freshwater fish are common, ~ 60 to 90% of people eat them every week [14].

The National Public Health Development Plan included an O. viverrini infection control program, and the five-year plan started its nationwide control operation from the sixth Health Development Plan (1987–1991). The national program contained three approaches based on focused prevention and control strategies, i.e., 1) mobile stool examination team providing case diagnosis and treatment, 2) hygienic defecation promotion to interrupt the transmission and 3) community preparation through mobilizing individuals, family members and community participation continuing effective health education focusing on avoiding the consumption of raw and undercooked fish dishes [15-17].

For decades, the national prevalence declined from 63.6% in 1987 to 9.4% in 2001. To date, the control activities continue only in target areas depending on problems and priorities in each community [11]. Thus, most target areas are located in the north and northeast, which have been recognized as endemic areas of O. viverrini infection. However, many reports showed that the prevalence was still high [16,17]. Uncooked fish preparations are ubiquitous and modifying traditional eating habits is difficult to achieve in general. Information regarding consumption of the more specific raw and undercooked fish dishes needs to be thoroughly studied to develop the more precise and sustainable interventions.

In 2002, we conducted a health survey in Baan Nayao Village, Sanamchaikaet District, Chachoengsao Province located in central Thailand and found that the prevalence of O. viverrini infection was 21.3%. The study population had migrated from the northeast and resettled to this area three decades ago. However, they have maintained their northeastern traditional life style including raw food consumption. From 2002–2004, a community-based cohort study was conducted to evaluate incidence and risk factors of O. viverrini infection. This previous study showed the incidence of 21.6/100 person-years and reported that Koi pla or chopped raw fish salad was the independent risk factor for O. viverrini infection [18]. Positive cases were treated with praziquantel and health education was provided to the community as suggested by the national liver fluke control program. From 2007–2009, the same population was followed up. Quantitative and qualitative techniques were used to re-assess the status of the infection in order to develop more effective intervention programs.

## Methods

The study comprised of quantitative and qualitative methods. From 2007–2009, the incidence and risk factors of acquiring O. viverrini infection were determined by a quantitative approach using a prospective cohort design comparable to our previous study carried out from 2002–2004 [18]. In 2009, the qualitative technique, focus group discussion (FGD), was used to explain the cause of unsatisfied infection rate in bio-psycho-social aspects.

### Study area

The study was conducted in Baan Nayao Village, Sanamchaiket District, a remote rural area in Chachengsao Province. The district is situated in the central region of Thailand, 120-km east of Bangkok. The village was isolated from the nearest central district and consisted of approximately 2,000 people. The majority of the population comprised of farmers who mostly retained their traditional northeastern culture, dialect, folk customs and life style including eating habits.

Concerning health-related services, a health promoting hospital was located at the center of the village covering basic primary care facilities for the whole population. The nearest district hospital is located a one-hour drive in Sanamchaiket District. Local village health volunteers were assigned for primary health care activities in the village.

### Quantitative study

#### Study design

The quantitative study was a community-based prospective cohort study. In 2007, a total survey for O. viverrini infection was conducted in this area. A total of 1,204 stool specimens were processed. We found that 224 persons (18.6%) were positive for O. viverrini infection at the baseline survey. Those who were negative for O. viverrini infection (n = 980, 81.4%) in the 2007 survey were invited to participate in a follow-up study in 2009 to measure the incidence and determine the risk factors. The follow-up time was 17 months.

#### Stool collection and examination

During the baseline and follow-up surveys, the same methods of stool collection and examination were performed. After a single stool specimen was collected from each participant, the examination process was then conducted in the field within six hours by two standardized methods, i.e., wet preparation and Kato technique [19]. The specimens were diagnosed for intestinal parasites under a light microscope by experienced examiners focusing on O. viverrini ova. These specimens were transported to the Department of Parasitology, Phramongkutklao College of Medicine, Bangkok for an additional examination method, i.e., formalin-ethyl acetate concentration technique [20]. At least one positive result from any of these three methods would be interpreted as positive result for O. viverrini infection.

During stool processing, the specimens were collected and registered into systematic code by the assigned co-researcher. The examiners were blinded to the specimen’s provider characteristics using a code-embedded procedure. Finally, the results were recorded to matched participants and transferred to the data management unit.

#### Questionnaires

Research participants were interviewed using standardized questionnaires to assess basic demographic and risk factor information. The questionnaires had been used in the previous cohort study and demonstrated an ability to identify potential risk factors for acquiring O. viverrini infection [18]. Each participant was registered with a systematic code corresponding to stool specimens. Risk assessment covered modes of transmission, eating habits such as a history of traditional fish dishes during the past 12 months, types of uncooked fish preparations and sources of fish.

The fish preparations were classified in three categories based on time of fish preservation. First, uncooked fish, chopped raw fish salad (Koi pla) in which fresh raw fish was chopped, mixed with other ingredients and then eaten instantly after preparing. This uncooked fish dish also included consumption of whole raw fish. Second, briefly fermented fish (Pla som) was preserved with salt three to seven days before eating. Third, extensively fermented fish (Pla ra) was preserved for six months in a highly concentrated salt solution [21,22].

#### Data analysis

The incidence density was reported as incidence rate per 100 person-years. All statistical parameters were calculated with a p value of 0.05 and 95% confidence interval using STATA/SE for Windows, Version 2. The associations between risk factors and O. viverrini infection were measured using Pearson’s chi-square for categorized variables. Risk factors were reported as relative risk. The crude relative risk (RR) was calculated by bivariate analysis. Multivariate analysis was performed using the Poisson regression method for adjusted relative risk.

### Qualitative study

The qualitative study used FGD technique [23] to assess: 1) situation of uncooked fish consumption in the community, 2) knowledge of O. viverrini infection, 3) attitude to O. viverrini infection, 4) uncooked fish consumption behaviors, 5) diagnosis and treatment of O. viverrini infection and 6) solution to O. viverrini infection. Participants were purposively selected and categorized into four groups. Each group comprised seven to eight members, i.e., 1) uninfected female group, 2) infected female group, 3) uninfected male group and 4) infected male group. The profiles of the participants were created based on information from questionnaires and stool examination results. The conversation during FGD was recorded using a voice recorder. The data was collected continuously until no new information was identified, i.e., data saturation.

The recorded conversations from each session were transcribed into text. Then text-based data were manually sorted and coded in order. Data were revised, organized and summarized for analysis. The methods used included content analysis, direct quotations and selected words to give consideration to actual local words used by the participants [24].

### Ethical consideration

The study protocol was reviewed and approved by the Ethics Committee of the Royal Thai Army Medical Department. Written informed consent was obtained from enrolled participants or parents of young participants following standard protocols. For those who tested positive for intestinal parasitic infection, the proper antiparasitic treatment was provided.

## Results

### Quantitative study

#### Characteristics of the study population

From 980 subjects who were negative for O. viverrini infection in the baseline survey in 2007, 793 were enrolled in the follow-up study in 2009, an 80.3% response rate. Characteristics of the 793 enrolled subjects and the prevalence of O. viverrini infection in each group is shown in Table 1. The prevalence of O. viverrini infection significantly differed by sex (p = 0.005) and age group (p = 0.019) while occupation had no significant difference. Regarding uncooked fish consumption behaviors, the percentages among the study population included consumption of chopped raw fish salad (48.1%), briefly fermented fish (39.9%) and extensively fermented fish (77.4%).

**Table 1:** Characteristics of participants and prevalence of Opisthorchis viverrini infection

**Characteristic**	**No. enrolled**	**No. infected (%)**	**p value**
Gender			
Male	364	105 (28.9)	
Female	429	85 (19.8)	0.005
Age group (years)	(Mean Age = 38.7, SD = 20.6)		
0 – 24	114	16 (14.0)	
25 – 59	288	78 (27.1)	
≥ 60	391	98 (25.1)	0.019
Occupation			
Agriculture	248	66 (22.6)	
Others	220	60 (27.3)	0.372
Total	793	192 (24.2)	

#### Incidence and risk factors of O. viverrini infection

The incidence of O. viverrini infection was 21.4/100 person-years. From Table 2, univariate analysis shows that males (RR = 1.5, 95% CI = 1.1-2.0), age group of 25–59 years (RR = 2.0, 95% CI = 1.1-3.3), and age group of 60 and older (RR = 1.8, 95% CI = 1.1-3.1) were statistically significant factors for acquiring the infection. For uncooked fish dishes, consumption of chopped raw fish salad (RR = 1.8, 95% CI = 1.2-2.6) was associated with O. viverrini infection, while the other dishes, i.e. briefly and extensively fermented fish, were not significantly associated.

**Table 2:** Univariate and multivariate analysis of risk factors for acquiring Opisthorchis viverrini infection

**Characteristics**	**Number of O. viverrini infected persons**	**Person-years of follow up**	**Incidence rate (/100 person-years)**	**Crude relative risk (95% CI)**	**Adjusted relative risk (95% CI)**
**Gender**					
Female	85	476.7	17.8	1	1
Male	105	407.0	25.8	1.5	1.3
				(1.1-2.0)	(0.9-1.8)
**Age group (years)**					
0-24	16	130.5	12.3	1	1
25-59	78	326.0	23.9	2.0	1.6
				(1.1-3.3)	(0.9-2.8)
60+	98	440.2	22.3	1.8	1.9
				(1.1-3.1)	(1.0-3.4)
**Occupation**					
Others	60	248.2	24.2	1	
Agriculture	66	282.8	23.3	1.0	-
				(0.7-1.4)	
**Fish menus**					
Chopped raw fish salad (Koi pla)					
No	50	294.0	17.0	1	1
Yes	80	262.7	30.5	1.8	1.6
				(1.2-2.6)	(1.1-2.3)
Briefly fermented fish (Pla som)					
No	25	96.8	25.8	1	
Yes	54	182.7	29.6	1.2	-
				(0.7-1.9)	
Extensively fermented fish (Pla ra)					
No	16	62.0	25.8	1	
Yes	64	217.5	29.4	1.1	-
				(0.7-2.1)	

95% CI = 95% confidence interval.

Adjusted for occupation, briefly fermented fish and extensively fermented fish.

In multivariate Poisson regression analysis, the age group of 60 years and older and consumption of chopped raw fish salad were independently associated with O. viverrini infection. Those who were 60 years of age and older were 1.9 (95% CI = 1.0-3.4) times more likely to acquire the infection than those under 25. Moreover, those who consumed chopped raw fish salad were 1.6 (95% CI = 1.1-2.3) times at risk of acquiring the infection, than those who did not consume chopped raw fish salad after adjusting for gender, age, occupation and history of consumption of other fish dishes.

### Qualitative study

#### Characteristics of the study population

A total of four groups were conducted for FGD consisting of 13 males and 17 females. The mean age of FDG participants was 28 for females (range: 22–65 years) and 38 for males (range: 30–58 years). All were literate, having attained the educational level of primary to junior high school. The occupation of the majority of FGD participants was as agriculturalists.

#### Status of raw fish consumption in the village

Of three uncooked fish dishes, the most frequently consumed dish was extensively fermented fish (Pla ra), since it was used as a core ingredient in north-eastern food. Chopped raw fish salad (Koi pla) was also a popular dish especially in the harvest season, i.e., October to December, when cyprinoid fish are considered more delicious. Pla som, briefly fermented fish, was consumed periodically.

Generally, main meals were prepared by the house maker. To date, Koi pla was not served as the main meal because they were aware of its negative consequences that would affect their children, spouse and themselves. However, Koi pla was prepared by males as a savory side dish for social drinking after work. Children and females were inclined to reduce consumption of uncooked fish dishes since the campaign by Phramongkutklao College of Medicine began in 2004. However, male adults seemed to respond less.

#### Perceptions of transmission of O. viverrini infection

Most of them knew that raw fish consumption could cause liver fluke infection. However, they did not know which type of raw fish dishes caused the infection. They all thought that both Koi pla and Pla ra could be the sources of infection. Moreover, some of them suspected that other aquatic creatures such as shrimp and vegetables could transmit the infection. A 45-year-old uninfected female unconvincingly stated:

“It may be in shrimp and we ate chopped raw shrimp salad so we got infected. Water plants in the pond gave us liver fluke infection too”.

There were common misconceptions with the route of transmission. Some villagers thought that eating any raw food, skin penetration or drinking water from natural sources could cause the infection. Some participants believed that mixing rice whisky (40% alcohol) or lime juice with raw fish dishes could kill the liver flukes.

#### Awareness of O. viverrini infection and raw fish consumption

Most participants knew that liver fluke infection was mostly asymptomatic or caused mild symptoms, so that villagers were not concerned about the infection. Cholangiocarcinoma was a fatal consequence but some participants, mostly those infected, did not realize the relationship between the disease and raw fish consumption. Other reasons the villagers continued consuming raw fish were that uncooked fish was delicious, convenient to prepare and it was a prudent meal since it needed no cooking equipment, fuel or fire. They insisted that the north-eastern (Isarn) people traditionally ate raw food. It would be hard to change their lifestyle. A 54-year-old infected male insisted:

“I think our environment forces us to eat this way. I eat the way our parents do. It’s inevitable”.

Some of them perceived that even after consuming uncooked fish dishes for a long period, they were still healthy and sometimes their stool examination produced negative results. In their insistence of raw fish consumption, they described that Koi pla and Pla som could be omitted for most females but Pla ra was impractical to be excluded.

#### Perceptions of treatment and prevention

The diagnosis of O. viverrini infection could not be achieved by the health promoting hospital. The villagers did not seek proper diagnosis and treatment since O. viverrini infection was usually asymptomatic. They knew that O. viverrini infection needed a specific antihelminthic drug, praziquantel. This drug was not available in the health promoting hospital or local drug stores. The medication was provided by Phramongkutklao College of Medicine. Most of the villagers used proper toilets at home; however, there were no latrines in the rice paddies where they worked. In addition, most immigrant workers defecated in the field because they did not stay in houses like local villagers.

#### Perceptions of health education and promotion

The villagers did not recognize O. viverrini infection as a health problem until the staff and medical students of Phramongkutklao College of Medicine conducted the campaign in 2004. After the liver fluke control campaign began, information of the disease was disseminated throughout the village. They suggested that the control campaign should be carried out regularly, and that community participation would take part in sustainability. They mentioned that they would stop eating if they knew it would cause cancer. A 35-year-old uninfected female stated:

“If we showed them the terrifying outcomes of the disease, they would stop eating raw fish. Cancer would be one of the most terrible consequences that they could imagine”.

They suggested that promoting the campaign among school children would yield a good result in the future. The children were the next generation and more open and adaptive to change their eating behaviors.

## Discussion

This study community was the first area where two cohort studies of O. viverrini infection were conducted over a five-year interval. Information from the cohort study would be useful for improved evaluation of intervention successes; however, to date it was rarely conducted in this context. In the present study, a qualitative technique was also performed. Again, qualitative information from real life intervention areas is very scarce. In fact, they are of tremendous importance to improve intervention programs. Some limitations of the present cohort series must be expressed. Since each cohort study was independently conducted, the results could not be directly comparable. However, it would be beneficial to consider the disease trend in the area. From the study design; using a similar methodology as the first cohort in 2002, negative cases were exclusively followed up for measuring the incidence. Therefore, the true disease burden might be underestimated because those who were positive from baseline study were excluded.

The study was conducted outside northeastern and northern regions of Thailand, the known endemic areas of O. viverrini. The community approach was employed resembling the national control strategy. The outcome showed that the incidence rate did not decrease when comparing 21.6/100 persons-year from 2002–2004 [18], with 21.4 /100 persons-year from 2007–2009 (Table 3). The number of newly infected villagers with O. viverrini each year indicated the effectiveness of the prevention program. Although the infected cases may be cured by chemotherapy, the new infections appeared at the same level. Compared with the previous study by Rangsin et al. (2009), the advantage of the present study was that we achieved a much higher response rate (80.3% vs. 60.3%) [18]. Both cohort studies used three methods to diagnose O. viverrini infection; one of them was the formalin-ethyl acetate concentration technique, considered to be highly sensitive and reliable to detect O. viverrini eggs [20]. Even so, when only one stool sample from each participant was examined, light infection could be missed. In addition, these microscopy-based methods could not clearly distinguish O. viverrini and small intestinal fluke eggs such as Haplochis. In 2004, Truab et al. (2009) showed that no small intestinal flukes were identified in this area using a PCR technique [25]. Therefore, we postulated that all Opisthorchis-like eggs identified in this study were O. viverrini. We used questionnaires to obtain food consumption history covering a one-year period; this could have produced a bias from the long recall period. As in our previous study, we obtained the exposure information at the same time as collecting stool specimens to reduced information bias [18].

**Table 3:** Comparison of the incidence and risk factors of Opisthorchis viverrini infection between 2 cohort studies in this area

	**Rangsin et al. **[18]	**The present study**
Study duration	2002-2004	2007-2009
Incidence (/100 person-years)	21.6	21.4
Significant risk factors (Adjusted RR, 95% CI)		
Age 20–39 years	3.1 (1.1-8.2)*	
Age 40–59 years	2.7 (1.0-7.4)*	
Age ≥ 60 years	4.1 (1.45-11.8)*	1.9 (1.0 – 3.4)**
Consuming Koi pla	1.9 (1.1-3.3)	1.6 (1.1-2.3)

*compared with 0–19 age group, ** compared with 0–24 age group.

Using multivariate analysis, the potentially independent risk factors for acquiring O. viverrini infection was consumption of chopped raw fish salad (Koi pla), while other uncooked-fish dishes were not significant factors. This risk factor was consistent with the previous study [18]. This information was substantially important because it could demonstrate the actual situation and effective solution. The age group of 60 years and older was statistically significant for acquiring O. viverrini infection; those who were older than 60 were 1.9 times more likely to get the infection compared with those who were less than 25. These results were also similar to our study conducted five years ago (Table 3).

From qualitative data, the villagers found it impractical to follow the unspecific raw fish consumption prevention campaign believing that they had to stop eating all of them, including extensively fermented fish (Pla ra), the main ingredient of north-eastern dishes. In fact, a few studies showed that metacercariae of O. viverrini could not survive the fermentation process of Pla ra, i.e., highly concentrated salt solution for six months [21,22,26]. It was consistent with our quantitative results showing that chopped raw fish salad (Koi pla), but not extensively fermented fish (Pla ra) was an independent risk factor for acquiring opisthorchiasis. Prevention and control programs should have a vast impact on reducing Koi pla consumption, not just a general term of uncooked-fish consumption. The clear message, “Avoid Koi pla Consumption” should be used among the villagers to prevent them from acquiring O. viverrini infection. This could make the health promotion campaign more feasible to practice.

From FGDs, male adults prepared and consumed Koi pla more often than others. This finding was similar to those reported in previous studies [27,28]. This dish was popular among male adults especially when they were drinking socially. Some misbelieves such as lime juice and alcohol could kill the liver fluke, were used as an excuse. Thus, health education and promotion targeting this group should be one of the main campaigns in the control program. We have been using the village health volunteer system to disseminate the information of O. viverrini infection. Regular and long term control programs can be facilitated by this system. Regarding school children, the villagers mentioned that they should be the primary target for health education since they can more easily change their habits. In addition, they can also deliver the knowledge and regularly remind their family about this disease. The role of children on prevention and control of O. viverrini infection should be further evaluated.

Improving diagnosis and treatment at the local health center should be performed as a national strategy because many north-eastern communities are distributed around the country. Most O. viverrini patients were asymptomatic; thus, active case detection should be performed especially where the prevalence of O. viverrini infection is high. Praziquantel was rarely available in both the local health center and drug stores. This prevented the villagers from taking it whenever they consumed raw fish. It has been indicated that praziquantel can cause the release of parasite antigens, which induces the recruitment of inflammatory cells [29]. This stress may cause pathologic condition and carcinogenesis. Adequate knowledge about levels of opisthorchiasis treatment have to be achieved before providing information to the community. Finally, the primary prevention of O. viverrini infection was preferred rather than the secondary prevention [30].

Unhygienic defecation occurred mostly while working in the rice paddies. Building facilities and proper use of latrines in the field should be considered by the community to prevent transmission. Both registered and unregistered migrants were working in the areas. Inadequate health care services for this population especially unregistered workers might be due to security, language and financial barriers. Unlike Thai villagers, these migrant workers usually lived in temporary shelters with no proper toilets. Prevention and control programs of O. viverrini infection should be set up for this population as well. Dogs and cats could be an important reservoir of O. viverrini [31]. Further studies should evaluate the role of these pets in this area.

## Conclusions

O. viverrini infection in the northeastern community residing outside of the northeast Thailand has been neglected since the national control program only covers well-known endemic areas, i.e., northeastern and northern Thailand. Although the conventional control program had been used in the study population, the incidence of O. viverrini infection remained the same. Continuation of raw fish consumption in this population was due to inadequate knowledge, misbeliefs, and social and cultural mores. In addition, unhygienic defecation, inadequate diagnosis and treatment also contributed to the infection. Our information could be used to improve the intervention so that it is more precise and effective. Specific raw fish dishes such as Koi Pla causing O. viverrini infection should be indicated in the control program. Specific risk groups such as social drinking males should be mainly targeted.

### Consent

Written informed consent was obtained from the patients for the publication of this report.

## Competing interests

The authors declare that they have no competing interests.

## Authors’ contributions

PS, MM, RR and SL contributed to the conception and design of the study. TN, MM and PS performed stool examination. PS, SK and MM collected and analyzed the data and wrote the manuscript. All authors read, revised and approved the final version that was submitted for publication.
